# Hereditary hearing loss SNP-microarray pilot study

**DOI:** 10.1186/s13104-018-3466-7

**Published:** 2018-06-14

**Authors:** Barbara Vona, Michaela A. H. Hofrichter, Jörg Schröder, Wafaa Shehata-Dieler, Indrajit Nanda, Thomas Haaf

**Affiliations:** 1 Institute of Human Genetics, Julius Maximilians University, Würzburg, Germany; 2 Department of ORL, Plastic, Aesthetic and Reconstructive Head and Neck Surgery, Comprehensive Hearing Center, Würzburg, Germany

**Keywords:** Copy number variation, Genotyping arrays, Hereditary hearing loss, Infinium HumanOmni1-Quad, Illumina, SNP-microarray

## Abstract

**Objectives:**

Despite recent advancements in diagnostic tools, the genomic landscape of hereditary hearing loss remains largely uncharacterized. One strategy to understand genome-wide aberrations includes the analysis of copy number variation that can be mapped using SNP-microarray technology. A growing collection of literature has begun to uncover the importance of copy number variation in hereditary hearing loss. This pilot study underpins a larger effort that involves the stage-wise analysis of hearing loss patients, many of whom have advanced to high-throughput sequencing analysis.

**Data description:**

Our data originate from the Infinium HumanOmni1-Quad v1.0 SNP-microarrays (Illumina) that provide useful markers for genome-wide association studies and copy number variation analysis. This dataset comprises a cohort of 108 individuals (99 with hearing loss, 9 normal hearing family members) for the purpose of understanding the genetic contribution of copy number variations to hereditary hearing loss. These anonymized SNP-microarray data have been uploaded to the NCBI Gene Expression Omnibus and are intended to benefit other investigators interested in aggregating platform-matched array patient datasets or as part of a supporting reference tool for other laboratories to better understand recurring copy number variations in other genetic disorders.

## Objective

Copy number variations (CNVs) are a well-recognized cause of genetic disease through the disruption of gene dosage and/or expression. However, their contribution to hereditary hearing loss (HL) has long been underestimated and remains an important question. Recently, a better appreciation of the CNV-burden in HL patients has emerged, with one study estimating that CNVs are implicated in up to 18.7% of patients in whom a genetic cause of HL was identified [1]. Ongoing efforts in the field are underway to not only diagnose patients, but also to identify genes underlying HL [2–4]. The high frequency of CNVs in Mendelian phenotypes such as HL support a SNP-microarray analysis strategy for the purpose of identifying chromosomal aberrations in known and candidate genes [5].

In this project, we ascertained HL patients in whom a molecular genetic diagnosis could not be determined from exclusionary *GJB2* (DFNB1A) screening to assess the contribution of CNVs to the diagnostic rate of HL. Our analysis established *STRC* (DFNB16) as a frequent cause of congenital HL [6] and identified a rare syndromic form of HL caused by a de novo deletion in the chromosome 4q35.1q35.2 region [7]. The data have also identified patients with inconspicuous SNP-microarray array findings who have advanced to projects that utilize high-throughput sequencing and bioinformatics analysis [8, 9]. The most interesting and impactful results from this work have been published. We have subsequently shifted our research efforts to employ whole exome sequencing in our HL cohort. However, we believe these SNP-microarray data may be of retrospective interest and offer valued information to the scientific community.

## Data description

### Patient recruitment

We studied the genomic DNA extracted from whole blood of 99 consecutively recruited patients with suspected hereditary HL and 9 unaffected family members between February 2011 and May 2013. Index patients with suspected environmental forms of HL were excluded. Family members were included, when possible, to enhance data analysis. Prior to investigation in this research setting, the patients had undergone routine diagnostic *GJB2* screening that included Sanger sequencing and duplication/deletion analysis using a multiplex ligation-dependent probe amplification approach. Patients with homozygous or compound heterozygous pathogenic *GJB2* variants were excluded from the study. In parallel, clinical records were collected and reviewed that are summarized in Data File 1 listed in Table 1. Data File 1 also includes familial relationships, if available.

**Table 1:** Overview of data files/data sets

Label	Name of data file/data set	File types (file extension)	Data repository and identifier (DOI or accession number)
Data set 1	Infinium HumanOmni1-Quad v1.0 SNP-microarray data	This dataset contains the raw intensity data files (Grn.idat and Red.idat) of each patient, as well as Matrix Signal Intensities (.txt) and Matrix Processed data (.txt)	NCBI Gene Expression Omnibus Data series accession: GSE111131 Data identifiers: GSM3022603, GSM3022604, GSM3022605, GSM3022606, GSM3022607, GSM3022608, GSM3022609, GSM3022610, GSM3022611, GSM3022612, GSM3022613, GSM3022614, GSM3022615, GSM3022616, GSM3022617, GSM3022618, GSM3022619, GSM3022620, GSM3022621, GSM3022622, GSM3022623, GSM3022624, GSM3022625, GSM3022626, GSM3022627, GSM3022628, GSM3022629, GSM3022630, GSM3022631, GSM3022632, GSM3022633, GSM3022634, GSM3022635, GSM3022636, GSM3022637, GSM3022638, GSM3022639, GSM3022640, GSM3022641, GSM3022642, GSM3022643, GSM3022644, GSM3022645, GSM3022646, GSM3022647, GSM3022648, GSM3022649, GSM3022650, GSM3022651, GSM3022652, GSM3022653, GSM3022654, GSM3022655, GSM3022656, GSM3022657, GSM3022658, GSM3022659, GSM3022660, GSM3022661, GSM3022662, GSM3022663, GSM3022664, GSM3022665, GSM3022666, GSM3022667, GSM3022668, GSM3022669, GSM3022670, GSM3022671, GSM3022672, GSM3022673, GSM3022674, GSM3022675, GSM3022676, GSM3022677, GSM3022678, GSM3022680, GSM3022681, GSM3022682, GSM3022683, GSM3022684, GSM3022685, GSM3022686, GSM3022687, GSM3022688, GSM3022689, GSM3022690, GSM3022691, GSM3022692, GSM3022693, GSM3022694, GSM3022695, GSM3022696, GSM3022697, GSM3022698, GSM3022699, GSM3022700, GSM3022701, GSM3022702, GSM3022703, GSM3022704, GSM3022705, GSM3022706, GSM3022707, GSM3022708, GSM3022709, GSM3022710, GSM3022711 GSE111131_Martix_signal_intensities.txt.gz GSE111131_Matrix_Processed.txt.gz
Data file 1	Data file 1: clinical overview Details: this table provides an overview of the audiological and clinical characteristics of each anonymized patient, as well as patient population background and familial relationships, if present	This file is available as an excel (.xls) table	NCBI Gene Expression Omnibus Data series accession: GSE111131 Data identifier: GSE111131_Data_file_1.xls.gz
Data file 2	Data file 2: sample sheet all Details: this table contains the sample sheet that includes the patient sex and anonymized ID with the beadchip position and barcode information and parental relationships, if present	This file is available as a comma separated variables (.csv) table	NCBI Gene Expression Omnibus Data series accession: GSE111131 Data identifier: GSE111131_Data_file_2.csv.gz

### Experimental protocols

The Illumina Infinium HD assay was performed according to manufacturer’s instructions using 200 ng genomic DNA. The Infinium HumanOmni1-Quad v1.0 SNP-microarrays (Illumina) were scanned using the BeadArray Reader and the iScan that are included in the last column of Data File 1 (Table 1).

## Data analysis

Unprocessed raw intensity data (.idat files) shown in Data Set 1 of Table 1 were generated. Additionally, raw and normalized green and red intensities (GSE111131_Matrix_signal_intensities.txt.gz), as well as matrix processed data (GSE111131_Matrix_Processed.txt.gz) were assembled. For our study, data were loaded into GenomeStudio v.2011.1 software and the B allele frequency and log R ratio were analyzed using Manifest H, cnvPartition 3.2.0, and QuantiSNP 2.2 [10]. The sample sheet that contains the necessary information to match the patient IDs with the sub-array data for this analysis are included in Data File 2 (Table 1).

## Limitations

This study was undertaken to initiate screening of a cohort of diagnostically unresolved HL patients. Of particular interest was obtaining a greater understanding of the contribution of CNVs to hereditary HL, which was underappreciated at the time of study initiation. As this was a pilot study, our intention was to screen a small cohort of 99 patients to gain insight into our primary research aims and then publish the most interesting findings separately [6, 7]. As our work has advanced to include high-throughput sequencing of genes involved in HL, it became evident that many clinically-relevant mutations reside beyond the resolution of the SNP-microarrays [8].

One further limitation relates to the clinical overview of the patients (Data File 1, Table 1). As this study was conducted between 2011 and 2013, any subsequent progression of HL or syndromes that may have manifested in patients after HL was diagnosed and clinical chart review occurred are not included. Thus, these data may not be well-suited for genome-wide association studies, but can nonetheless be included in data collections investigating other disorders with the disclaimer that these disorders, especially adult-onset disorders in patients who were recruited as children, cannot be conclusively excluded.

Technical limitations well-known to SNP-microarrays involve the inability to detect balanced translocations, copy-neutral alterations, and inversions that may nonetheless be relevant [11] for the clinical diagnosis of HL [12, 13].

## Abbreviations

CNV: Copy number variation
HL: Hearing loss
